# Fibrils from Designed Non-Amyloid-Related Synthetic Peptides Induce AA-Amyloidosis during Inflammation in an Animal Model

**DOI:** 10.1371/journal.pone.0006041

**Published:** 2009-06-30

**Authors:** Per Westermark, Katarzyna Lundmark, Gunilla T. Westermark

**Affiliations:** 1 Rudbeck Laboratory, Department of Genetics and Pathology, Uppsala University, Uppsala, Sweden; 2 Department of Clinical and Molecular Medicine, Linköping University, Linköping, Sweden; 3 Department of Medical Cell Biology, Uppsala University, Uppsala, Sweden; Massachusetts General Hospital and Harvard Medical School, United States of America

## Abstract

**Background:**

Mouse AA-amyloidosis is a transmissible disease by a prion-like mechanism where amyloid fibrils act by seeding. Synthetic peptides with no amyloid relationship can assemble into amyloid-like fibrils and these may have seeding capacity for amyloid proteins.

**Principal Findings:**

Several synthetic peptides, designed for nanotechnology, have been examined for their ability to produce fibrils with Congo red affinity and concomitant green birefringence, affinity for thioflavin S and to accelerate AA-amyloidosis in mice. It is shown that some amphiphilic fibril-forming peptides not only produced Congo red birefringence and showed affinity for thioflavin S, but they also shortened the lag phase for systemic AA-amyloidosis in mice when they were given intravenously at the time of inflammatory induction with silver nitride. Peptides, not forming amyloid-like fibrils, did not have such properties.

**Conclusions:**

These observations should caution researchers and those who work with synthetic peptides and their derivatives to be aware of the potential health concerns.

## Introduction

The amyloidoses comprise a large group of diseases in which abnormally folded proteins self-assemble into beta pleated sheet fibrils and form mainly extracellular deposits. More than 25 different human proteins are known as main amyloid fibril proteins [1]. In each disease there is only one specific amyloid fibril protein. In one of the disorders, secondary or reactive amyloidosis, a protein named AA is deposited as fibrils in most tissues, leading to a life-threatening condition mainly by deposits in the kidneys. Protein AA is derived from a plasma precursor, serum AA (SAA) by removal of a C-terminal part of the protein [2]. Plasma SAA is an acute phase reactant, synthesized by the liver as response to interleukin (IL) -1, IL-6 and tumor necrosis factor α. At an acute inflammation, the plasma concentration of SAA can increase as much as 1,000 times and reach as high as 1 mg/ml (1 g/liter) [3].

AA-amyloidosis is a consequence of a chronic inflammatory disease with persistently high plasma concentration of SAA. Infectious diseases like tuberculosis and leprosy are known causes of AA-amyloidosis although rheumatic diseases, particularly rheumatoid arthritis, are more commonly found to be complicated by AA-amyloidosis in the western world [4]. Only a fraction of patients with those conditions does, however, develop AA-amyloidosis. The reason for this is unknown.

SAA is a conserved protein and AA-amyloidosis is commonly found in many mammalian and avian species, including mouse (*Mus musculus*). Like human, mouse acute phase SAA is a product of two closely related genes resulting in SAA1 and SAA2. In the mouse, only SAA 1 is amyloidogenic [5]. Murine AA-amyloidosis can occur spontaneously but can also be induced by an inflammatory challenge. Typically, such amyloidosis appears after several weeks of persistent inflammation. This long indolent phase can be shortened drastically by administration of a small amount of an extract of amyloid from an already amyloidotic mouse [6], [7]. The nature of the active principle in the extract, called ‘amyloid enhancing factor’ (AEF) has been a matter of long discussions but recent studies indicate that it is the amyloid fibril itself acting as a seeding agent for amyloid fibril elongation [8]. In addition, the murine systemic apolipoprotein A-II-derived amyloidosis exhibits a similar mechanism of transmissibility [9].

Formation of amyloid-like fibrils can easily be mimicked *in vitro* with many natural or synthetic peptides which may spontaneously assemble into beta sheet fibrils with typical amyloid properties, including affinity for the dye Congo red and green birefringence after such staining [10]–[13]. Typically, this occurs after a lag period under which fibrils are not detectable [14]. Seeding a peptide solution with a small amount of preformed fibrils of the same kind dramatically reduces the lag period [15]. Already small differences in amino acid sequence can greatly reduce nucleation efficacy [16] but cross-nucleation may occur with amyloid-like fibrils of a biochemical composition different from that in the solution [17].

We have shown earlier that synthetic amyloid-like fibrils from some short peptides corresponding to segments of amyloid fibril proteins may have AEF-activity in the experimental AA-amyloid mouse system [18], [19]. Such fibrils, when given intravenously at the induction of AA-amyloidosis with silver nitrate, reduced the lag phase before AA-amyloidosis developed. Although the exact mechanism by which the synthetic fibrils worked is unknown, it was shown that the first AA-amyloid developed exactly where the synthetic fibrils could be traced in tissues. It is therefore likely that the fibrils acted as direct seed on which AA-fibrils developed. Although amyloid always is a pathological deposition in human, there are amyloid-like fibrils occurring normally in nature such as silk and bacterial curli. Also such fibrils can exert AEF-effect [20].

Self-assembling peptides constitute a potentially very valuable tool for biomaterials, which can be used for different purposes, e.g. as scaffolds for tissue repair [21]. Different strategies are used and one is to construct peptides which assemble into stable β-sheet structures that, under certain circumstances can form fibrils and, consequently, may be amyloid-like. In the present study we have investigated the amyloidogenic properties of a group of designed synthetic peptides. The peptides were further studied for their ability to enhance AA-amyloidosis in mice.

## Materials and Methods

### Synthetic peptides

Peptides used in this study were commercially synthesized (Synpep, Dublin, CA) and a kind gift from Dr. Shuguang Zhang, MIT, Cambridge, MA. The purity of the peptides was evaluated by reversed phase high performance liquid chromatography and the expected masses were confirmed with mass spectroscopy.The peptides and their intended structures are shown in Table 1.

**Table 1:** Synthetic peptides used in the study of amyloid-fibril like properties.

Peptide No.	Amino acid sequence	Structure	Amyloid-	like fibrils	in vitro
			Congo red*	Thioflavin S**	EM***
1.	Ac-DDDDAARRRR-amide	α-helix	-	-	Amorphous
2.	Ac-GGGGDD	surfactant	-	-	Amorphous
3.	Ac-VVVVVVDDDD	surfactant	-	-	Sheets, no fibrils
4.	Ac-AAAAAADDDAAAAAA-amide	surfactant	-	-	Small rodlike structures
5.	Ac-AAAAAAKK-amide	surfactant	+	+	Thin, long, curvy fibrils
6.	DAAAAAAR	α-helix	++	+	Thin, long fibrils
7.	EEEEAAAAAAKKKK	α-helix	(+)	(+)	Thin, curvy fibrils
8.	Ac-VVVVVVD	surfactant	-	-	Paper-like sheets
9.	Ac-VVVVVVDD	surfactant	-	-	Paper-like sheets
10.	Ac-AAAAAAD	surfactant	-	-	Thin uneven fibrils
11.	Ac-FKFEFKFE-amide	β-sheet	++	++	Long, parallel fibrils
12.	Ac-RADARADARADARADA-amide	β-sheet	++	++	Heterogeneous bundles of thin fibrils

* The affinity for Congo red and green birefringence in polarized light was semiquantitatively estimated. -, no reaction; (+) partial weak reaction; + weak affinity and green birefringence. ++ strong affinity and bright green birefringence.

** The strength of fluorescence after labeling with thioflavin S was semiquantitatively estimated. (+) partial weak reaction; + weak fluorescence; ++ strong bright fluorescence.

*** Electron microscopy after that the peptides had been dissolved in dimethyl sulfoxide, diluted in water and incubated for 1 month.

### Fibril formation

The ability of the peptides to form amyloid-like fibrils was tested with a standard method [22]. Peptides were dissolved at a concentration of 20 mmol/l in dimethylsulfoxid (DMSO) (stock solution). Aliquots were diluted 1∶10 with distilled water to 2 mmol/l and incubated at room temperature without shaking. Small aliquots (0.8 µl) were removed at different time points (5 min, 24 hours, 1 week, 2 weeks, 1 month, 3 months, 1 year), placed on glass slides, air dried and stained with alkaline Congo red for examination in polarized light [23]. The one-year-old material was tested for binding of thioflavin S. Dried droplets were stained as described [24], mounted in water-based mounting media and investigated in a fluorescence microscope (exitation 440 nm and emission 488 nm). After one month, small aliquots were also taken for electron microscopy. These were diluted further 1∶10 in distilled water, applied on formvar-coated copper grids, dried and contrasted with 1% sodium phosphotungstate and viewed in a Jeol 1200 electron microscope operated at 80 kV.

Peptide No. 12 was also available as preformed 1% hydrogel (PuraMatrix, BD Biosciences, San Jose, CA) and had been dissolved directly in water.

### Amyloid induction

Outbreed female NMRI mice were obtained from B & K Universal, Södertälje, Sweden. After an inflammatory challenge with silver nitrate subcutaneously, this strain of mice develops AA-amyloidosis after >5 weeks [8]. The mice were 6–8 weeks old at the beginning of the experiments and had free access to water and pellets (type R 36; Lactamin, Vadstena, Sweden). The induction of AA-amyloidosis has been described in detail earlier [8]. Shortly, at day 0, under anesthesia groups of 7–8 animals were given 0.1 mg synthetic fibrils in 0.1 ml in 10% DMSO intravenously, followed by 0.3 ml 1% silver nitrate subcutaneously in the back. Since addition of peptide monomers on seeded fibrils takes place at short ends, fibril suspensions were treated with a burst of ultrasound prior to injection in order to create fibril fragments. For all animal groups, the injections of silver nitrate were repeated on day 7 and 14 and the animals were sacrificed on day 16.

In a second set of experiments, a prolonged amyloid induction protocol was used. Peptides, now including some of them not forming amyloid-like fibrils, were dissolved in DMSO and diluted with water as described above. After 1 month of incubation in room temperature, 0.1 mg of the material was given intravenously and 0.3 ml 1% silver nitrate subcutaneously in the back to groups of 7 mice. Further injections of 0.1 ml 1% silver nitrate were given subcutaneously after 1, 2 and 3 weeks and the animals were sacrificed on day 23.

Since the properties of fibrils might change after long-term maturing, a third series of mice were tested will all peptides after they had incubated as above for 1 year. The prolonged protocol (23 days) was applied.

In a separate experiment peptide No. 12 was dissolved directly in distilled water at 1% concentration, instantly forming a fibrillar network [21], [25]. After a short tip-sonication, 0.1 mg was injected intravenously in 12 mice and silver nitrate was given according to the prolonged amyloid induction protocol.

**Figure 1:**
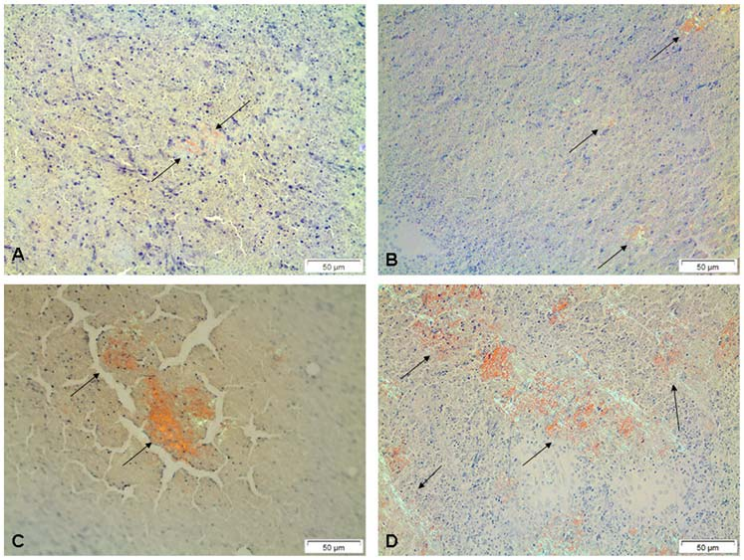
Semiquantitative estimation of amyloid deposits in splenic squash preparations. A, grade 1. Only one or two amyloid particles are found in the slide. B, grade 2. Several small amyloid deposits are found. C, grade 3. More widely spread amyloid deposits often with large particles appear. D, grade 4. Extensive amyloid material all over the slide. Amyloid deposits are marked by arrows. Bar = 50 µm.

For both protocols (16 and 23 days) groups of control mice received inflammatory challenges identical to the experimental groups but got only vehicle intravenously. In all the experiments one half of the removed spleen was squashed between two microscopical slides, air-dried and examined in a polarization microscope for the presence of amyloid after that specimens had been stained with alkaline Congo red [23]. This method allows examination of more of the organ than after paraffin-embedding and sectioning. For the animals, given one-year-old material, the amount of amyloid was semiquantitatively estimated according to the following scale: 0, no amyloid found; 1+, one or two amyloid-containing fragments/slide; 2+, more than two amyloid fragments/slide but no coalescent areas; 3+, amyloid-containing fragments with coalescence; 4+, extensive amyloidosis. Examples of the four different amyloid grades are given in Fig. 1.

The animal studies were approved by the Local Animal Ethic Committee at Linköping University, Linköping District Court, PO Box 365, SE-58103, Linköping, Sweden.

## Results

### Aggregation properties of peptides

All peptides were easily soluble in DMSO. After dilution with water to 10% DMSO, gel particles were seen within minutes with peptide No. 11. These where strongly congophilic and showed bright green birefringence in polarized light. The other solutions stayed clear. The diluted preparations were incubated for up to 12 months, aliquots were taken for study of amyloid-like materials at several time-points and occurrence of amyloid-like material was checked by Congo red staining of dried droplets. Congophilia and birefringence, a hallmark of amyloid and typical of cross-beta fibrils had appeared after 1 week for peptides No. 5, 6, 7, 11 and 12, (Table 1) but with somewhat different appearances. The solution with peptide No. 7 had a precipitate that had affinity for Congo red stain but exhibited only weak green birefringence. Peptides No. 6, 11 and 12 all showed typical amyloid appearance after staining with Congo red with a strong green birefringence at polarization microscopy. No congophilic materials developed in the other solutions ever after incubation for 12 months.

**Figure 2:**
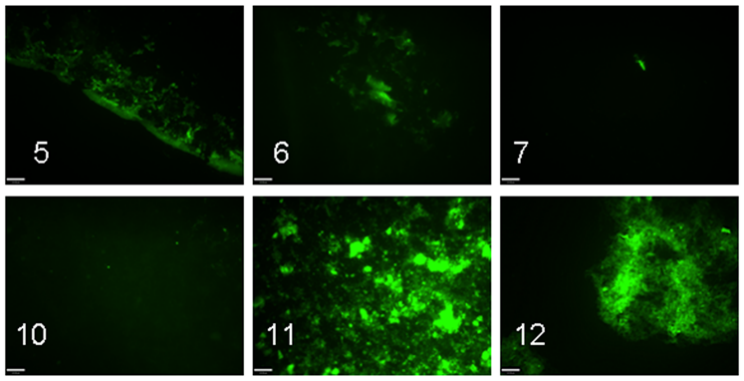
Small drops of aggregated synthetic peptides were dried on microscopical slides, labeled with thioflavin S and examined in a fluorescence microscope. The figure shows that peptides No. 5, 6, 7, 11 and 12 exhibited affinity for the dye and fluorescence. Peptide No. 10, although fibrillar by electron microscopy, did not show this amyloid-like property. Bar = 24 µm.

Fluorescence after labeling with thioflavin S strikingly paralleled the findings with Congo red (Table 1 and Fig. 2). Thus, thioflavin S fluorescence was only seen with material from peptides 5, 6, 7, 11 and 12. Particularly strong labeling was seen with peptides 11 and 12 (Fig. 2).

**Figure 3:**
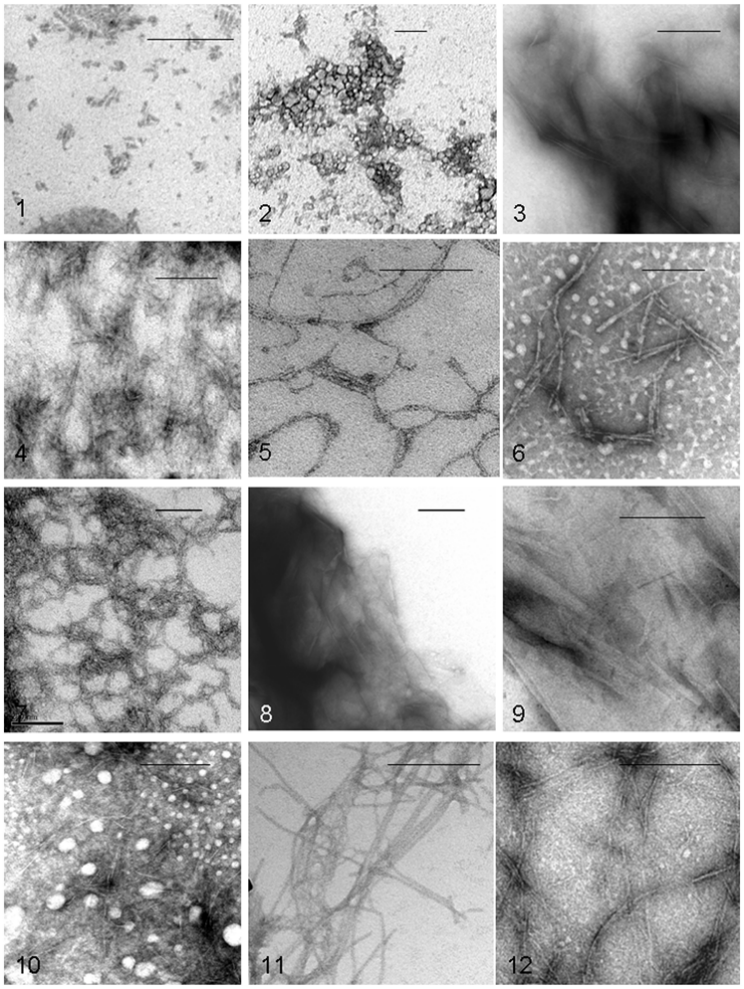
All aggregated peptides, listed in Table 1 were examined in transmission electron microscopy. Peptides No. 4, 5, 6, 7, 10, 11 and 12 formed fibrillar assemblies while peptides No. 3, 8 and 9 gave rise to paper-like sheets. Bar = 200 nm.

Using electron microscopic examination, (Fig. 3) notable differences were seen between the different peptide preparations but a variation was also apparent with the same peptides. Peptide No. 5 (AAAAAAKK) gave rise to thin (3–4 nm), relatively long fibrils, often aligned laterally to tape-like structures up to 20 nm in width. Some short fibrils also appeared. In contrast peptide No. 6 (DAAAAAAR) showed about 8 nm thick, slender fibrils of varying length with little tendency to aggregate laterally. Peptide No. 7 (EEEEAAAAAAKKKK) consisted of curvy, rope-like structures in which about 5 nm thick fibrils were aligned parallel but also small, straight fibrils occurred. Fibrils from peptide No. 11 (FKFEFKFE) were very long, slender and even with a diameter of about 10 nm. They were possibly composed of two very closely aligned parallel filaments. No short fibril fragments were seen. Material from peptide No. 12 (RADARADARADARADA) exhibited a highly heterogeneous morphology. The basic structure seemed to be thin filaments of less than 3 nm width, arranged in irregular bundles of varying length. Peptides No. 3, 8 and 9 showed thin, flat, somewhat paper-like arrangements.

### Effects of fibrils on induction of amyloidosis in mice

**Figure 4:**
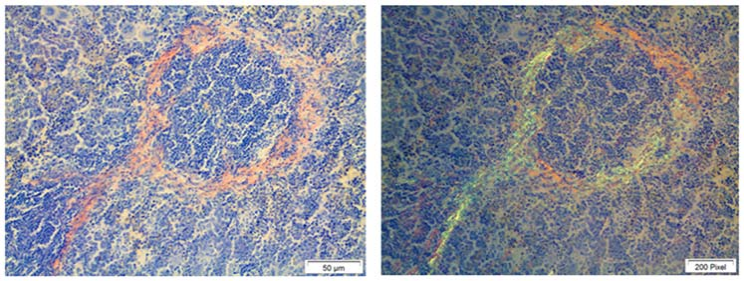
Amyloid deposits perifollicularly in the spleen. Typically, this is the first region where systemic AA amyloid is seen in the mouse. Bar = 50 µm.

In the first series (16 days), the ability of amyloid-like fibrils 5, 6, 7, 11 and 12 to enhance the development of murine AA-amyloidosis was tested. Administration of the different peptide fibrils intravenously had no apparent direct effects in the mice. At the end of the experiment, squash preparations of spleen (Fig. 1), which is the initial site for AA-amyloid deposition (Fig. 4), were scrutinized for the presence of amyloid by an investigator not knowing the origin of the material. Amyloid was found in the spleen of three animals, two of which had received peptide No. 11 and one with peptide No. 12 (Table 2). One of the mice in group 12 died during the experiment.

**Table 2:** Effects of peptides on the development of AA-amyloidosis in mice.

Peptide No.	Amino acid sequence	Duration	of	inflammation	Grade£
		16 days$	23 days$	23 days*	
1.	Ac-DDDDAARRRR-amide	N/A	0/7#	0/6#	0
2.	Ac-GGGGDD	N/A	0/7	0/5	0
3.	Ac-VVVVVVDDDD	N/A	N/A	0/6	0
4.	Ac-AAAAAADDDAAAAAA-amide	N/A	N/A	0/8	0
5.	Ac-AAAAAAKK-amide	0/7	1/7	2/7	1,2
6.	DAAAAAAR	0/7	2/6	1/5	1
7.	EEEEAAAAAAKKKK	0/7	0/7	0/7	0
8.	Ac-VVVVVVD	N/A	N/A	1/8	1
9.	Ac-VVVVVVDD	N/A	N/A	0/6	0
10.	Ac-AAAAAAD	N/A	N/A	0/8	0
11.	Ac-FKFEFKFE-amide	2/7	0/7	3/7	1,1,2
12.	Ac-RADARADARADARADA-amide	1/6	4/7	5/6	1,1,1,1,4
13.	Ac-RADARADARADARADA-amide	N/A	0/12	N/A	N/A

$ diluted peptide solutions matured for 1 month.

* diluted peptide solutions matured for 1 year.

£ amyloid grade semiquantitatively estimated according to 0, no amyloid found; 1+, one or two amyloid-containing fragments/slide; 2+, more than two amyloid fragments/slide but no coalescent areas; 3+, amyloid-containing fragments with coalescence; 4+, extensive amyloidosis. The amyloid grade of each positive mouse is given.

# Number of mice with amyloidosis/total number of animals. Several animals had to be taken out from the experiment due to development of skin ulcers at the site of silver nitrate injection. None of these animals had developed amyloid deposits.

N/A, not applicable.

Peptides 1–12 were dissolved in dimethyl sulfoxide and then diluted in water. Peptide 13 was directly dissolved in water.

Given the low number of amyloidotic mice with the 16 day protocol the rest of the experiments were prolonged to 23 days. Amyloid developed in 1 and 2 animals given peptide No. 5 and 6, respectively. Of the 7 mice given peptide No. 12, 4 animals developed splenic amyloid deposits (Table 2). This experiment also included peptides 1 and 2. None of the animals administered with these peptides showed any evidence of amyloid deposition.

All of the peptide materials were matured for 1 year and then tested for their ability to accelerate induction of AA-amyloidosis. As seen in Table 2, particularly peptide No. 12 was effective in this respect. Interestingly, when effect of fibrils with amyloid properties (fluorescence with thioflavin S and affinity for Congo red and subsequent green birefringence; peptides 5, 6, 7, 11 and 12) was compared with that of aggregates without these properties (peptides 1, 2, 3, 4, 8, 9 and 10), it was evident that the amyloid-like fibrils were significantly more amyloid-accelerating than the others (p<0.001, Fisher's exact test). However, quantitative estimation of the amount of deposits in amyloid-positive animals did not reveal any apparent difference between the peptides (Table 2).

In the control group of animals only receiving vehicle intravenously in addition to the inflammatory stimulus, no amyloid was detected in any animal. The group of animals that received peptide No. 12 as a preformed hydrogel combined with silver nitrate subcutaneously was also free of amyloid.

## Discussion

Amyloidosis is a group of devastating disorders in which proteins or peptides undergo conformational changes and undertake an abnormal β-sheet structure, leading to assemblies into highly ordered thin filaments which, in turn, arrange parallel, often with a twist, to the final protease-resistant fibrils. The seeding mechanism acts by addition of new monomers to the short ends of the preformed fibrils. Whether this occurs with monomers that already have adopted an amyloid-prone conformation or the preformed fibrils induce such structure in molecules is not known.

Seeding *in vitro* is protein-specific and already small differences in amino acid sequence can reduce the efficacy significantly [16], [17]. However, cross-seeding may occur *in vitro* and it has been shown that Aβ fibrils efficiently seed human IAPP [17]. The dramatic reduction in time for the development of experimental AA-amyloidosis *in vivo* by amyloid fibrils either given intravenously or orally is also believed to occur by a seeding mechanism. The cross-seeding phenomenon, shown *in vitro*, obviously also can take place *in vivo*. Thus, the time for the development of AA-amyloidosis can be greatly shortened not only by extracted fibrils from another animal (i.e. AEF) but also to a certain degree by amyloid fibrils of other biochemical composition [26], (Westermark et al., unpublished), by synthetic fibrils from segments of known amyloid fibril proteins [18], [19] and by naturally occurring, amyloid-like fibrils such as *Bombyx mori* silk, Sup35 or curli from *E. coli* [20], [27]. Similar findings have been obtained with the apolipoprotein A-2 murine model of systemic amyloidosis [9], [28], [29].

Some of the peptides (peptides No. 6 and 7), designed to adopt α-helix conformation, assembled into amyloid-like fibrils with affinity of Congo red and green birefringence, typical of repeated β-sheet structure. However, motives in several of the known amyloid fibril proteins, including the prion protein [30], [31] and protein AA [32], transform from a native α-helical state into β-sheet conformation in amyloidogenesis.

The present study shows that some fibrils, made from synthetically designed peptides, with amyloid-properties including affinity for Congo red with concomitant green birefringence in polarized light, also may enhance AA-amyloidosis in the murine experimental model. Thus, 4 out of 5 amyloid-like peptide-fibrils had some effect *in vivo*. The apparent efficacy varied and the most amyloidogenic peptide seemed to be RADARADARADARADA when fibrils were made by dilution of the peptide initially dissolved in DMSO. Interestingly, when the same peptide formed fibrils after being dissolved directly in water, no amyloidogenic properties were noted in the used system. Such fibrils did not bind Congo red and showed no green birefringence in polarized light. Obviously the same peptide can obtain fibrillar structures with different properties. It has been found that the morphology and properties of seeded fibrils depend on the seed and that different metastable morphologies can be obtained with one and the same peptide [33], [34]. The seeding mechanism it is not understood but it is possible that subtle variations in secondary and tertiary structure of an amyloid fibril protein can alter the efficacy of cross-seeding and that a perfect fit at critical regions is necessary for a monomer to dock. Given the finding that not every mouse developed amyloidosis with any of the synthetic fibrils, such exact fit in cross-seeding may be rare and perhaps transient.

These experiments were carried out in a mouse model of systemic AA-amyloidosis and nothing is known regarding the possible initiation by environmental factors of the human counterpart. However, it is by no way ruled out that human amyloid diseases also can be initiated by a seeding mechanism, either by natural homologous fibrils or by fibrils from animals [35]. In addition, as is shown in this work, it is necessary to be aware of the possibility that also designed synthetic peptide fibrils may introduce new risk factors. One such risk is that fibrils may start protein misfolding and aggregation, leading to amyloid diseases.
